# The Policy of Waiting

**Published:** 1919-08-16

**Authors:** 


### The Hospital

The Workers' Newspaper of Administrative Medicine and Institutional Life, Administration, National Insurance and Health.

No. 1732, Vol. LXVI. SATURDAY, AUGUST 16, 1919. PRICE TWOPENCE

### THE POLICY OF WAITING.

Although the peace signing is an accomplished fact, there is still an abundance of signs that problems of demobilisation, or, more accurately, the return of the public to peace-time routines, will take no little time for their solution. There are a thousand exasperating situations with us at present, and, apart from the continued epidemic of strikes, it is maddening to the person who simply wants some work done, but is told there is no one to do it, and hears daily of "out of works" and people in receipt of unemployment pay. For many medical men, the position presents undreamed-of difficulties. There is still the oft-repeated trouble that, for those returning from active service, the practices which they had formerly built up have dwindled considerably in their absence; we will not reopen the discussion, for, as a matter of fact, it is probable that as the country becomes more settled, there will be ample opportunity for all medical men who so desire to enter, with reasonable prosperity, into general practice.

A more noticeable and important development is the desire of so many demobilised officers from the Army and Navy to avoid this branch of the profession, whether they have previously experienced it or not. It may be that the expense of housekeeping, the difficulties of even finding a house and the element of social uncertainty have turned the attention of the younger men towards Government posts with fixed salaries which, except in the case of gross professional mistakes, reduce the chances of failure to a minimum. Probably it is this feeling of social and financial insecurity which is keeping many men waiting for the very large number of Government medical posts which must very soon be created, rather than any definite scorn or dislike for general practice as a scientific and interesting career. It seems that these doctors soon must be provided with the opportunity they desire and many of them are now, as seen by the large entry lists for the higher examinations, rightly attempting to improve their qualifications to the maximum extent. Whatever form the new medical services take, there will undoubtedly be needed a large number of whole and part-time salaried medical officers; while the tuberculosis and venereal questions are certain to call for yet more.

Such circumstances readily explain the public impression that there is still a shortage of doctors in the country. They see in the profession yet another example of a class which on the one side seems to be deficient in numbers, and yet on the other a good deal unemployed. Certainly, a large number of these latter are now obtaining posts with the Ministry of Pensions. One hears that up to the present, the Ministry has been utilising the services of something like 3,000 medical men on the various pensions boards, while another 1,000 are officially employed outside the boards, in medical supervision of the disabled. Recently, to these officers it was explained that a large number of demobilised doctors were desirous of obtaining these posts and that in readjustment of appointments, the principle should and will undoubtedly be followed of giving preference to those men who have been on active service. Thus, in the future one may expect yet more openings to occur in this particular branch of service, but though, at present, a most satisfactory type of office, we cannot expect the length of tenure or prospects attached to them to be very great, for it is to be hoped that the problem of disabled men will not remain with us for any long period. Restoration to useful citizenship is already rapidly occurring, and one sees many signs of organised continued treatment for these men and it appears unlikely that, ultimately, more than a small proportion will be needed of the doctors at present occupied in the cure of our crippled and weakened soldiers.

Here it is not our object either to approve or criticise the action of the number of doctors waiting for the official developments of the future, but simply to point out the existence of this factor in medical unsettlement. In the new scheme gradually being evolved and launched by a number of old-established medical institutions for purposes of either research or improved teaching, it is more than possible that with the present indefinite appointments and low rates of remuneration, many of the men now taking up active posts under these organisations will, when the details of the new services are published, inevitably leave the older institutions, and that at a period possibly fatal to the perfect development of these well-meant plans of improvement. One reads of Government grants which should perhaps modify this tendency, but nevertheless, if it is to be expected to elaborate the working of our school and medical units in such a way as to produce steady and permanent upward progress, not only should men be now secured who are considered worthy of long association with the schemes themselves, but also their position must be made of such relative security that the whole personnel will not be scattered by coming announcements from the Ministry. Not only the doctors but more than a few great hospitals are, perhaps advisedly, adopting the waiting policy.

